# Seeing the unseen: illuminating *Toxoplasma gondii’s* metabolic manipulation

**DOI:** 10.1128/mbio.01211-24

**Published:** 2024-07-12

**Authors:** Diego Huet, Victoria Jeffers

**Affiliations:** 1 Center for Tropical and Emerging Global Diseases, University of Georgia, Athens, Georgia, USA; 2 Department of Pharmaceutical and Biomedical Sciences, University of Georgia, Athens, Georgia, USA; 3 Department of Molecular, Cellular and Biomedical Sciences, University of New Hampshire, Durham, New Hampshire, USA; University of Arizona, Tucson, Arizona, USA

**Keywords:** host-pathogen interactions, metabolism, apicomplexan parasites, confocal microscopy

## Abstract

Intracellular infection by a pathogen induces significant rewiring of host cell signaling and biological processes. Understanding how an intracellular pathogen such as *Toxoplasma gondii* modulates host cell metabolism with single-cell resolution has been challenged by the variability of infection within cultures and difficulties in separating host and parasite metabolic processes. A new study from Gallego-Lopez and colleagues (G. M. Gallego-López, E. C. Guzman, D. E. Desa, L. J. Knoll, M. C. Skala, mBio e00727-24, 2024, https://doi.org/10.1128/mbio.00727-24) applies a quantitative imaging approach to evaluate the host cell metabolism during intracellular infection with *Toxoplasma*. This study provides important insights into host metabolic responses to *Toxoplasma* infection and offers a valuable tool to dissect the mechanisms underlying parasite infection and pathophysiology.

## COMMENTARY

The metabolic responses of host cells to intracellular pathogens can reveal insights to mechanisms of pathology, parasite dissemination, and transmission. However, studying the metabolic flux of host cells during intracellular infection has been challenging due to difficulties in distinguishing between the metabolic outputs of host and pathogen. Furthermore, the dynamics of metabolism are rapid and require precise measurements in single live cells over the course of intracellular infection. A new study has harnessed a powerful imaging technique to measure metabolic responses in the host cell during infection with *Toxoplasma* tachyzoites (1). Using optical metabolic imaging (OMI), the authors accurately quantify and track reactive oxygen species and metabolite dynamics over the course of tachyzoite infection of human foreskin fibroblasts (HFFs).

OMI relies on the fluorescent properties of FAD and NAD(P)H and the differences in the lifetime of the free and protein-bound FAD and NAD(P)H in the cell (2, 3). Using fluorescence lifetime imaging microscopy (FLIM), Gallego-Lopez and colleagues quantified the proportions of the free and protein-bound forms of these metabolic cofactors, providing insight into the redox state of the host cell over the course of parasite infection. Compared to larger scale analyses, this non-invasive, label-free approach allows for high-resolution analysis of host metabolic responses in single cells, distinguishing between infected and uninfected cells and importantly, distinguishing between the infected host cell and the intracellular parasite. With this approach, they found that fibroblasts displayed a shift in redox balance with a significant increase in oxidation over the course of infection. From detailed analysis of intracellular levels of glucose and lactate, and metrics of glycolytic flux (glycolytic activity, capacity and reserve), they conclude that the increase in oxidation is likely due to changes in host metabolic dynamics that are influenced by intracellular parasite infection, such as a decrease in host cell glycolysis and a reduction in intracellular lactate levels.

This change is likely a parasite-initiated phenomenon, and not just a general host response to intracellular infection; host cells that undergo a chemically induced interaction known as “kiss and spit” in which the parasite attaches to the host membrane and secretes parasite effectors into the host cytoplasm but does not complete invasion were also observed to undergo similar metabolic changes. This observation underlines the power of this imaging technique in precisely quantifying metabolic changes in infected and uninfected cells close to infected cells (bystanders) within the same culture dish to fully understand the host responses to parasite infection. Interestingly, the metabolic responses in infected host cells did not appear to be influenced by the ability of the parasites to recruit the host mitochondria to the parasitophorous vacuole membrane. Parasite in which the mitochondrial association factor MAF1 was deleted were still capable of inducing fluctuations in host glycolysis and basal mitochondrial respiration, further implicating secreted parasite factors such as rhoptry proteins in altering host cell metabolism.

While this study examined only metabolic responses during infection of HFFs, the study establishes a proof of principle, opening the door to assess metabolic rewiring in other cell types, particularly those more relevant to *Toxoplasma* in a physiological context, such as macrophages, myocytes, and neurons. For instance, imaging *Toxoplasma*-infected macrophages, which are known to generate NAD+ *de novo* (4), may offer interesting insights into how *Toxoplasma* induces immune cells to aid in parasite dissemination (5, 6). As for myocytes and neurons, they primarily rely on oxidative phosphorylation and produce a high baseline optical redox ratio of FAD/[NAD(P)H + FAD], which makes them also amenable for optical metabolic imaging. Visualizing the metabolic rewiring in myocytes and neurons might help us understand the pathophysiology of *Toxoplasma* myocarditis and encephalitis. Furthermore, host cell hypoxia causes a switch from oxidative phosphorylation to glycolysis to produce sufficient ATP, and lactate dehydrogenase converts NADH and pyruvate into NAD+ and lactate. This reaction is critical for NAD+ regeneration and continuous glucose breakdown, which reduces the optical redox ratio (due to the rise in free cytosolic NADH) in the cells. OMI could, therefore, provide insights into how *Toxoplasma* infection alters host cell metabolism under low-oxygen conditions.

By combining cell morphology with intrinsic fluorescence, OMI could be used to simultaneously monitor metabolic changes in multiple cell types infected with *Toxoplasma*. The redox state of the host cell has previously been reported to regulate parasite development between acute and chronic stages of infection (7); thus, this technique could also be employed to determine—and maybe even predict—whether redox and ROS changes in host cells can influence *Toxoplasma* conversion from the acute to the chronic stage.

Finally, the application of OMI could be used to investigate how other intracellular eukaryotic and bacterial pathogens manipulate their host cell metabolism. The metabolic rewiring of parasites such as *Trypanosoma cruzi, Leishmania* spp., the liver stages of *Plasmodium* spp., *Mycobacterium* spp., and *Salmonella* spp. could be visualized, and perhaps different “metabolic manipulation signatures” obtained depending on the pathogen. In summary, this study validates OMI as a valuable tool for investigating the distinct metabolic changes in both host and microbe during intracellular infection that allow for successful dissemination and pathogenesis.
